# The nature and prevalence of disability in a Ghanaian community as measured by the Language Independent Functional Evaluation

**DOI:** 10.11604/pamj.2013.14.103.2142

**Published:** 2013-03-15

**Authors:** Benjamin William Kelemen, Andrew John Haig, Siera Goodnight, Gifty Nyante

**Affiliations:** 1 Johns Hopkins School of Medicine, USA; 2 The University of Michigan Department of Physical Medicine and Rehabilitation, USA; 3 The University of Ghana

**Keywords:** Rehabilitation, disability, Africa, epidemiology, literacy, health care policy

## Abstract

**Introduction:**

The current study uses the Language Independent Functional Evaluation (L.I.F.E.) to evaluate disability in a smaller Ghanaian coastal town to characterize the extent and nature of disability. The L.I.F.E. is a video animated, language free equivalent of the standard 10-item verbal/written Barthel Index functional assessment.

**Methods:**

Over a four-month period, the L.I.F.E. survey was given to members of the village of Anomabo in a preliminary survey which consisted of recruitment in an un-controlled manner, followed by a systematic, comprehensive survey of three neighborhood clusters. Basic demographics were also collected, along with the observer's assessment of disability.

**Results:**

541 inhabitants (264 in the preliminary survey and 277 in systematic survey) completed the L.I.F.E. Participants ranged from 7-100 years old (mean age 32.88, s.d. 20.64) and were 55.9% female. In the systematic study, 16.6% of participants had a less than perfect score on the L.I.F.E., indicating some degree of impairment. Significant differences were found between age groups, but not between sexes, the preliminary and systematic survey, and study location (a=.05).

**Conclusion:**

The L.I.F.E. and this study methodology can be used to measure the prevalence of disability in African communities. Disability in this community was higher than the frequently cited estimate of 10%. African policymakers can use the L.I.F.E. to measure disability and thus more rationally allocate resources for medical rehabilitation.

## Introduction

The personal and economic burden of disabling medical illnesses across the world is unquestioned. The World Disability Report is often cited to state that 10% of the world population lives with a disability [1]. However resources to manage disability are not equitably allocated. Among developing countries, it is estimated that only 2% of people with disability receive any rehabilitation whatsoever [2]. In Africa there are only 6 physician specialists in Physical and Rehabilitation Medicine, whereas regions such as China have up to 10,000 specialists [3].

The reasons for misallocation of resources are many and complex. However one probable reason is the difficulty in obtaining information on the extent and cost of disability in low-resource regions. This is an especially challenging problem for African epidemiologists and policy makers, who must deal with hundreds of languages and relatively high illiteracy rates.

Recently a tool for measuring disability without the use of language has been designed. The Language Independent Functional Evaluation (L.I.F.E.) is a video pictorial representation of the functions portrayed in the commonly used Barthel Index [4, 5]. After preliminary designs were tested in the United States and Ghana, a final version has demonstrated good content and face validity [6], and has been shown equivalent or superior to the Barthel Index in a Mongolian community [7].

The current study brings the L.I.F.E. back to Africa to evaluate disability in a smaller Ghanaian coastal town. Results may shed light on the extent and nature of disability in such a community, but also demonstrate the viability of this relatively high-tech approach to the problem in a low-resource region.

## Methods

**Figure 1:**
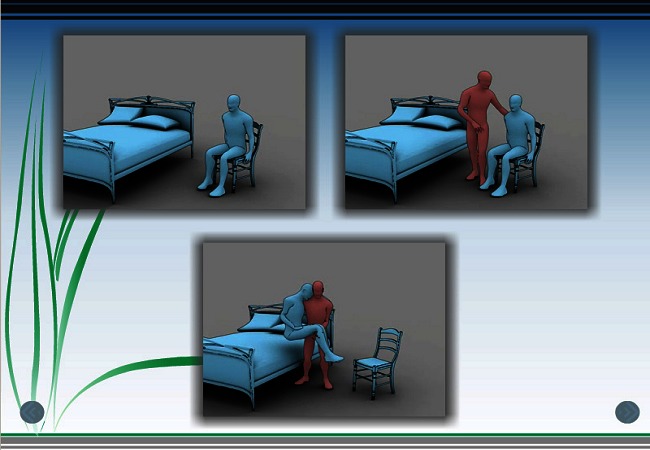
Still pictures from the video animated by L.I.F.E. Portrayal of chair and bed transfer ©2008 the regents of the University of Michigan reproduced with permission

The L.I.F.E. has been described in detail previously [4, 7]. Instructions to the subject consist of a very short video of a person in a wheelchair choosing his method of ambulation (a wheelchair) from among 4 choices on a computer screen. They are accompanied by a brief recorded instruction in the person's native language, introducing the nature and purpose of the testing. Subsequently, without any words or language symbols, the subject is shown a sequence of picture groups that represent the 10 functions also represented in the Barthel Index. These include feeding, bathing, grooming, dressing lower body, bowel continence, bladder continence, transfer to toilet, cleaning after toilet, transfer bed to chair, ambulation (wheelchair or walking), and stair climbing. Each picture group typically includes persons performing the task unassisted, with some assistance, and completely dependent in the task. The subject touches the screen over the picture that describes his or her ability and goes to the next group. Figure 1 is an illustration of one L.I.F.E. screen.

In a previous study the summary L.I.F.E. scores have been shown to relate well to the Barthel Scores (Spearman's rho=0.757, p.75), except bowel and bladder, which had moderate correlations [7].

The Ghanaian coastal village of Anomabo was chosen because it is familiar to the various investigators. Anomabo is situated directly on the main highway between Accra and Cape Coast. It has a well organized local government health care system with a clinic in both central Anomabo and its close neighbor Biriwa. The village of Anomabo itself has a population of roughly 3,000. Because of its location on the main highway yet also on the ocean front, the town's main economy is split between fishing and tourist attractions. Nearly half of the men in the village seem to be fisherman, while there are also three different hotels and guest houses in Anomabo itself. Although the official language of Ghana is English, most of the villagers speak Fante, so the introduction and instructions were recorded in Fante and played before the L.I.F.E. was given.

The project was performed over a four month period. Surveys were performed by an American (BWK) who lived in the community for the duration of the study. He had cursory knowledge of the Fante language, and was provided with extensive guidance on local culture and community from the local Ghana Health Service nurse.

To test the functionality of L.I.F.E. the investigators first used an uncontrolled open method of subject recruitment, basically approaching people on the street and near the clinic. Subsequently a more specific methodology was used, in which the investigators comprehensively surveyed neighborhoods in regions referred to in this paper as “Rural West North”; Rural West South, and Town Center.

The investigators surveyed three main large clusters, two from the western corner and one from the center of town. Because the town, particularly on the outskirts, is not very clearly organized in terms of streets, geographic landmarks were used to delineate the borders of the clusters.

The first cluster extended from the western border of Anomabo past the beach resort and the road attached to the beach resort and all the way to the next cluster of houses as an eastern border. The main highway and the ocean made up the North and the South borders respectively.

The second cluster began on the northern side of the highway at the western border of Anomabo. It extended in the East all the way to the row of houses just past the opposing entrance to the Beach Resort. In the North it extended back until there seemed not to be any houses left that had been completed. In the West it extended to the Western border of Anomabo, just past the gas station.

The third cluster was in the center of town. The center of Anomabo extends South from the main road towards the beach. There are three main streets extending away from the Accra-Cape Coast road. The length of the westernmost of these streets all the way from the Accra-Cape Coast road to its southernmost dead-end were surveyed. We defined occupants of houses as permanent habitations. Thus vendors who occasionally slept in their store along the street were not included in the cluster survey.

Households living on selected streets or paths in these regions were all included. If a person was away or a house was empty, the investigator returned in subsequent days to ensure participation. With subject consent, the survey began with an informal assessment as to whether the person interviewed had an obvious disability, using information and criteria obvious to the public. (In order for the study to be exempt from the ethical review no medical history or physical examination was permitted). The subjects performed the L.I.F.E., and were dismissed.

Data was transferred to an excel spreadsheet for further analysis. Data from the initial informal survey was separated from the more stringent survey, but is also reported. Statistical analyses were performed with SPSS software (version 17.0, SPSS Inc., Chicago). Descriptive statistics were tabulated, along with one-sample Kolmogorov Smirnov (K-S) tests which showed non-parametric distribution of scores for participants. Spearman's rho correlation coefficient, Mann Whitney-U Test, and Kruskall-Wallis tests were conducted to assess the relationships between disability (total L.I.F.E. scores) and demographic factors (a=.05).

Approval was obtained from the Ghana Health Service, and the local authorities, and the study was formally exempted from review by the American university's ethical review board.

## Results

The L.I.F.E. and demographics were collected for a total of 541 individuals residing in the village of Anomabo. The preliminary survey consisted of 264 inhabitants and the final survey, which included the areas of Rural West North, Rural West South, and the Town Center, was comprised of 277. In the final survey, 55.9% of participants were female, with ages ranging from 7-100 years old (mean age 32.88, s.d. 20.64). Out of these, 66.5% of participants had received some formal education. Table 1 provides more details of participant demographics.

**Table 1:** Subject Demographics

	Preliminary Survey	Final survey	Rural West North	Rural West South	Town Center
**Age (mean ± s.d.)**	28.26±17.78*	32.88±20.64	33.67 ± 19.22	34.26 ± 20.68	40.96 ± 23.84*
**Age Distribution**	7-99	7-100	12-78	12-95	12-100
**Sex (% female)**	54.5	55.9	68.9	48.5	59.7
**Education**					
**None**	n/a	33.5	22.2	21.2	46.3
**Primary school**	n/a	26.5	28.9	24.2	27.6
**Secondary school**	n/a	35.3	48.9	43.4	24.6
**Beyond secondary school**	n/a	4.7	0	11.1	1.5
**% with observable disability**	6.1	3.9	0	2	2.2
**% with any disability on L.I.F.E**.	16.3	16.6	13.3	19.4	16.4
**L.I.F.E. Total Score**					
**(mean, s.d.)**	18.65 ± .99	18.48 ± 1.81	18.80 ± .66	17.80 ± 3.45	18.54 ± 1.45

* Mean age of preliminary survey differed significantly from final survey areas

* Mean age of town center differed significantly from mean age of Rural West South

**Figure 2:**
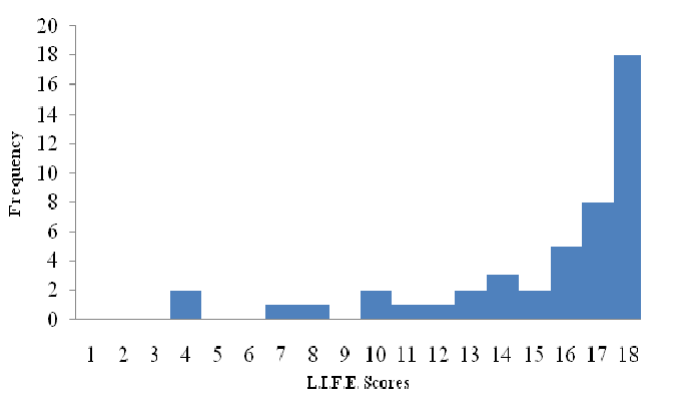
Distribution of total L.I.F.E in scores in final survey.

In this community 16.6% of participants provided a less than perfect score on the L.I.F.E., indicating some degree of impairment. The average L.I.F.E. score in the final survey was 18.48 (s.d. 1.81). Figure 2 shows that a few people had profound multifactorial disability while many others had a few disabilities. The nature and extent of functional limitations is outlined in detail in Table 2. Stair climbing, walking mobility and bowel continence were the most common single disabilities.

**Table 2:** The nature and extent of disability among 277 persons participating in the final survey

	Individual Function L.I.F.E Scores				
Functions	0	1	2	3	Any Disability
	N (%)	N (%)	N (%)	N (%)	N (%)
Feeding	1 (.4)	4 (1.4)	272 (98.2)	-	5 (1.8)
Bathing	6 (2.2)	271 (97.8)	-	-	6 (2.2)
Grooming	15 (5.4)	262 (94.6)	-	-	15 (5.4)
Dressing	7 (2.5)	5 (1.8)	265 (95.7)	-	12 (4.3)
Bowel continence	9 (3.2)	1 (4.0)	257 (92.8)	-	20 (7.2)
Bladder continence	8 (2.9)	6 (2.2)	263 (94.9)	-	14 (5.1)
Transfer onto toilet	11 (4.0)	266 (96.0)	-	-	11 (4.0)
Cleaning after toileting	11 (4.0)	266 (96.0)	-	-	11 (4.0)
Bed to chair transfers	5 (1.8)	7 (2.5)	265 (95.7)	-	12 (4.3)
Wheelchair/walking mobility	4 (1.4)	4 (1.4)	9 (3.2)	260 (93.9)	17(6.1)
Stair climbing	4 (1.4)	16 (5.8)	257 (92.8)	-	20 (7.2)
Total L.I.F.E. score	-	-	-	-	47 (17.0)

* The final column ‘any disability’ represents persons with all but the highest score for each function.

**Figure 3:**
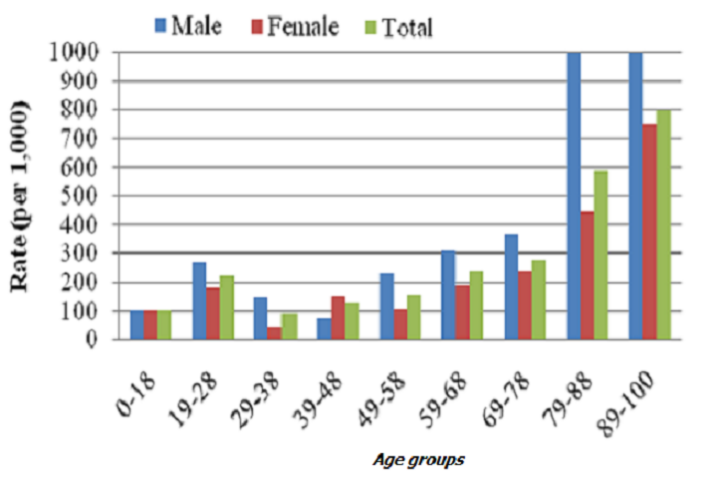
Prevalence of disability as measured by the L.I.F.E by age and gender

The relationship between age and disability is complex, as outlined in Figure 3. To evaluate the differences in L.I.F.E. scores between age groupings (0-18, 19-28, 29-38, 39-48, 49-58, 59-68, 69-78, 79-88, 89-100), a Kruskal-Wallis test was conducted. The test, corrected for tied ranks, was significant; X^2^ (8, N=272) =42.13, p=.000. The proportion of variability in the ranked dependent variable accounted for by study location was .156, indicating moderate relationship between L.I.F.E. scores and age. Post-hoc tests were conducted to evaluate pair-wise differences across groups, controlling for Type I error across tests by using the Bonferroni approach. The results of these tests are shown in Table 3.

**Table 3:** Pair-wise differences in total L.I.F.E. scores across age groups using Mann-Whitney U Test

Age Groups Z scores	0-18	19-28	29-38	39-48	49-58	59-68	69-78	79-88
0-18	-	-	-	-	-	-	-	-
19-28	−1.654	-	-	-	-	-	-	-
29-38	−0.286	−1.136	-	-	-	-	-	-
39-48	−0.839	−1.844	−0.985	-	-	-	-	-
49-58	−0.630	−0.559	−0.358	−1.202	-	-	-	-
59-68	−0.594	−0.462	−0.330	−1.211	−0.026	-	-	-
69-78	−3.334*	−1.729	−2.592*	−3.002*	−1.819	−1.593	-	-
79-88	−4.536*	−2.996*	−3.685*	−4.402*	−2.837*	−2.556*	−1.364	-
89-100	−3.969*	−2.674*	−3.415*	−3.924*	−2.673*	−2.488*	−1.614	−0.672

* Indicates significance (a<.05)

An independent samples Mann-Whitney U test was employed to examine the differences in L.I.F.E. scores in the final survey for the sexes. There were no significant differences in scores by sex z= -.176, p=.860. When examined by final survey location, Rural West North approached significance, z= -1.746, p=.081, while Rural West South and Town Center did not, z= -.670, p=.503 and z=1.006, p=.314, respectively.

From the researcher's informal observations, 3.9% of participants appeared to have an impairment. This is in contrast to the 16.6% of participants who scored some disability on the L.I.F.E. While Table 4 shows that there is a relationship between informal observation and formal survey, the substantial under-representation on casual observation is of note.

**Table 4:** The relationships between disability (L.I.F.E. total scores) and demographic factors in the Final Survey using non-parametric statistics (Spearman's correlation, Mann-Whitney U Test, Kruskall-Wallis Test)

	N	Spearman's Correlation	Z	X^2^	df	p-value
**Age**	272	−0.236				0.000*
**Sex**	277		−0.176			0.860
**Education**	277			12.129	8	0.146
**Surveyor's informal Assessment of Function**	277			6.759	2	0.034*
**Final Survey Locations**	277			1.509	2	0.470

* Indicates significance (a=.05)

One goal is to determine whether convenience sampling is sufficient in comparison to the more arduous task of systematic survey. For the systematic survey there was no statistically significant difference in the crude rate of disability between the areas sampled. (Rural Northwest 133.3/1000, Rural Soutwest 193.9/1000, Town Center 164.2/1000, average 169.7/1000; (a>05) No significant difference in any disability as measured by the L.I.F.E. between preliminary survey and final survey locations. A Kruskal-Wallis test conducted to evaluate differences in the % of people disabled between survey settings, corrected for tied ranks, showed no significant differences X^2^ (3, N=541) =.915, p=.822. Table 5 describes the distribution of L.I.F.E. scores by area.

**Table 5:** Distribution of L.I.F.E. scores by area

LIFE Scores	Preliminary	Rural West North	Rural West South	Town Center	Final Survey
19 (no disability)	221 (83.7%)	39 (86.7%)	79 (80.6%)	112 (83.6%)	230 (83.0%)
17-18	33 (12.5%)	5 (11.1%)	8 (8.2%)	13 (9.7%)	26 (9.4%
15-16	7 (2.7%)	1 (2.2%)	2 (2.0%)	4 (3.0%)	7 (2.5%)
13-14	2 (0.8%)	-	1 (1.0%)	4 (3.0%)	5 (1.8%)
11-12	1 (0.4%)	-	2 (2.0%)	-	2 (.8%)
0-10	-	-	6 (6.1%)	1 (.7%)	7 (2.6%)

A Kruskal-Wallis test was conducted to evaluate differences in median age among study settings (Preliminary, Rural West North, Rural West South, and Town Center). The test, which was corrected for tied ranks, was significant; X^2^ (3, N=536) =32.461, p=.000. The proportion of variability in the ranked dependent variable accounted for by study location was .061, indicating a weak relationship between study location and participant age. Follow-up tests were conducted to evaluate pair-wise differences across groups, controlling for Type I error across tests by using the Bonferroni approach. The results of these tests indicated a significant difference between the preliminary survey and all three locations of the final survey, along with a significant difference in age between Rural West South and Town Center. Participants in the preliminary survey were significantly younger than in the final survey locations, along with those participants in the Town Center being significantly older than those in the Rural West South.

## Discussion

The study results show 17% of inhabitants of this rural African village have varying degrees of disability as measured by the L.I.F.E. in the final survey, and that the computer animated L.I.F.E. is accepted as a survey method among rural Africans. Further it showed that a systematic survey results in some differences compared with a convenience sample in the community, and that casual observation is grossly insufficient to detect most disability found with the L.I.F.E. Specific findings are worth comment and the methodology should be further discussed.

The population studied is not intended to reflect all of Africa, but rather the type of community in which accurate data on disability might be difficult to obtain. Nevertheless, the distribution of age, sex, educational levels, and absence of rehabilitation services are likely not different from other rural communities. This does not represent all disabilities. The L.I.F.E. does not measure communication or cognitive functions, for instance. To the extent Anomabo represents other African communities, the 10% number frequently cited by national governments is a gross underestimate of the prevalence and cost of disability. The relationships between function and basic demographics, as noted in Table 4, are pertinent. Older people were more disabled than younger people. However sex and education levels were not associated with level of disability.

It is not surprising that mobility related disabilities were found most commonly. Difficulties in bowel and bladder function were next most common, however it is important to note that the psychometric properties of surveys for these functions are typically not good, and the L.I.F.E. is no exception [4, 7]. Also the relative values and weights given to function can be challenged. All global standards for functional assessment include social assumptions that may not be valid. For example, in Anomabo, a community with few stairs, functions such as water carrying or boat launching may be more associated with societal participation and quality of life.

Qualitatively, the investigators observed significantly less functional disability than they had expected. Subjects with obvious disabilities often answered negatively with regard to their own function. In fact, subject's self-assessment of disability seemed to be associated most with increasing age and family support. The conclusion to be drawn from these counter-intuitive observations is not that the poverty and lack of medical care in Anomabo is somehow protective against disability. Rather, the lack of self-reported disability points to the fact that, in Anomabo, disability is most often fatal. Persons who cannot get themselves out of bed will not get out of bed; there is little support to help them with their disability beyond their own ingenuity and resistance.

The need to systematically survey disability is illustrated by some of the differences between our preliminary study and the final protocol. There were not drastic differences in percentage of persons with disability between the early and formal survey. However there were age differences. Whether the area of the preliminary survey was inhabited more by younger people or whether younger people were more ‘on the move’ and accessible to the convenience sample, can be debated. On the other hand it may be quite reasonable to believe that older people live closer to the center of town, as found here.

Overall the findings of this paper point to a more significant burden of disability than expected. Incontrovertible evidence points towards the effectiveness and cost-effectiveness of medical rehabilitation in reversing disability [3]. To date in Africa there has been exceedingly little investment in this special area. There are few training programs for physical therapists, occupational therapists, prosthetists, and other rehabilitation allied health programs; only a handful of multidisciplinary rehabilitation facilities, and essentially no Physical Medicine and Rehabilitation physicians.

The second finding of this paper-that a high-tech solution can be practical in measuring disability in rural, non-English speaking populations, provides an important solution. African governments and policymakers can use the L.I.F.E. to measure the burden of disability in their communities. The results can lead to rational allocation of resources — undoubtedly an increase in investment in medical rehabilitation.

## Conclusion

The L.I.F.E. and this study methodology can be used to measure the prevalence of disability in African communities. Disability in this community was higher than the frequently cited estimate of 10%. African policymakers can use the L.I.F.E. to measure disability and thus more rationally allocate resources for medical rehabilitation.
